# Predictive Coding Over the Lifespan: Increased Reliance on Perceptual Priors in Older Adults—A Magnetoencephalography and Dynamic Causal Modeling Study

**DOI:** 10.3389/fnagi.2021.631599

**Published:** 2021-04-09

**Authors:** Jason S. Chan, Michael Wibral, Cerisa Stawowsky, Mareike Brandl, Saskia Helbling, Marcus J. Naumer, Jochen Kaiser, Patricia Wollstadt

**Affiliations:** ^1^Institute of Medical Psychology, Goethe-University, Frankfurt, Germany; ^2^School of Applied Psychology, University College Cork, Cork, Ireland; ^3^Brain Imaging Center, Goethe-University, Frankfurt am Main, Germany

**Keywords:** sound-induced flash illusion, aging, multisensory integration, dynamic causal modeling, magnetoencephalography, transfer entropy, predictive coding, beta-band activity

## Abstract

Aging is accompanied by unisensory decline. To compensate for this, two complementary strategies are potentially relied upon increasingly: first, older adults integrate more information from different sensory organs. Second, according to the predictive coding (PC) model, we form “templates” (internal models or “priors”) of the environment through our experiences. It is through increased life experience that older adults may rely more on these templates compared to younger adults. Multisensory integration and predictive coding would be effective strategies for the perception of near-threshold stimuli, which may however come at the cost of integrating irrelevant information. Both strategies can be studied in multisensory illusions because these require the integration of different sensory information, as well as an internal model of the world that can take precedence over sensory input. Here, we elicited a classic multisensory illusion, the sound-induced flash illusion, in younger (mean: 27 years, N = 25) and older (mean: 67 years, N = 28) adult participants while recording the magnetoencephalogram. Older adults perceived more illusions than younger adults. Older adults had increased pre-stimulus beta-band activity compared to younger adults as predicted by microcircuit theories of predictive coding, which suggest priors and predictions are linked to beta-band activity. Transfer entropy analysis and dynamic causal modeling of pre-stimulus magnetoencephalography data revealed a stronger illusion-related modulation of cross-modal connectivity from auditory to visual cortices in older compared to younger adults. We interpret this as the neural correlate of increased reliance on a cross-modal predictive template in older adults leading to the illusory percept.

## 1. Introduction

Predictive coding theory suggests that our perceptual experience is determined by a fine balance between internal predictions based on priors acquired over the course of our lives and incoming sensory evidence (Arnal and Giraud, 2012; Wolpe et al., 2016). Sensory evidence and priors are thought to be fused in a Bayesian way, to arrive at a posterior representing the best guess regarding the state of the world, that produces our perception. Aging research offers an opportunity to probe this suggestion, as the amount of information accumulated in our priors increases throughout one's lifetime, while the precision of unisensory evidence degrades at later stages in life. The first factor will strengthen the influence of predictions, while the second reduces the influence of unisensory evidence. Together, they should tip the balance to a state, where perception is increasingly dominated by predictions. Investigating this change in balance is possible using perceptual illusions that arise when predictions take precedence over sensory evidence, such as the sound-induced flash illusion (SiFi).

The SiFi is the perception of two visual flashes when only one flash is indeed presented along with two auditory beeps, in relatively short succession (Shams et al., 2000; Shams and Beierholm, 2010; Hirst et al., 2020; Keil, 2020). This illusion occurs because the auditory modality has a higher temporal acuity compared to the visual system (Colavita, 1974; Ernst and Banks, 2002; DeLoss et al., 2013), thus we instinctively rely on cross-modal predictions generated by the auditory system (Setti et al., 2011). Older adults perceive more SiFi across a wider temporal binding window (TBW) compared to young adults (Colavita, 1974; Ernst and Banks, 2002; Setti et al., 2011; DeLoss et al., 2013; McGovern et al., 2014). This is because older adults are more likely to integrate multisensory stimuli compared to young adults (Laurienti et al., 2006; Stevenson et al., 2018). This was originally thought of as beneficial, as it effectively compensated for the loss of unisensory acuity (de Boer-Schellekens and Vroomen, 2014). However, it can also have a detrimental effect, as older adults integrate more sensory information across a longer time span, irrespective of its relevance to a task, compared to younger adults (Setti et al., 2011).

To date, the neural underpinnings as to how older adults integrate more multisensory information over time are far from understood. However, following the line of argument of predictive coding, it is possible that older adults rely on increased top-down “template” information (i.e., priors) compared to young adults. Perceptual illusions rely on such perceptual “templates” of our environment, which are ultimately violated, but still take precedence. The internal “template” relevant for the SiFi is that the more temporally reliable auditory event should be accompanied by a visual object—thus, when one flash is presented accompanied by two beeps, an additional visual object is inserted, and perceived (Kayser and Shams, 2015; Rohe and Noppeney, 2015). Wolpe et al. (2016) investigated the role of age on sensorimotor predictions using a force-matching task. They found that older adults over-compensated their grip-force when they attempted to directly resist an applied force. This over-compensation was greater than in young adults performing the same task. Critically, this difference between age groups disappeared when they were asked to match the force using a “slider” that was not directly connected to the applied pressure. Indeed, both groups were more accurate in the slider condition. Wolpe et al. (2016) suggested the increased over-compensation is due to older adults having greater sensory attenuation accompanied by an increase in the weighting of the efferent information.

If the predictive coding account is correct in its relation to aging and multisensory integration, then (i) illusions based on predictions should become more frequent with age. (ii) The neurophysiological signature of the illusory perception should also be found as a general marker when comparing young vs. older participants. (iii) Due to the predictive nature, this signature should be found preceding the illusion, and (iv) it should be found in the beta-band—according to recent neurophysiological accounts of predictive coding (Bastos et al., 2012; Brodski-Guerniero et al., 2017). (v) The neurophysiological correlates of the aging process should manifest as network effects in terms of information transfer and effective connectivity, where brain areas generating reliable predictions should increase their influence over other brain areas that deliver less precise sensory evidence. Indeed, previous SiFi studies in young adults have demonstrated increased pre-stimulus beta-band activity for trials when an illusion was perceived compared to no illusion (Keil et al., 2014; Kaiser et al., 2019, see also Lange et al., 2013; Keil and Senkowski, 2018; Hirst et al., 2020).

There are a number of factors that can affect the integration of sensory information, such as age (Laurienti et al., 2006; Freiherr et al., 2013), impairments (Setti et al., 2011; Chan et al., 2015), and familiarity with the stimuli (Setti et al., 2011). Additionally, a number of factors including the number of stimulus onset asynchronizes can also affect the rate of perceived illusions (Chan et al., 2018). Indeed, within each of these studies there are also individual differences to the rate of perceived illusion which are averaged across. In the following study, we stratified participants within each of the age groups into those with a propensity to perceive the illusion and those without a propensity to perceive the illusion.

To assess the neurophysiological correlates of illusory perception and effects of aging, we used magnetoencephalography (MEG) combined with beamformer source reconstruction. For the analysis of network effects, we used a novel combination of information theory, in particular transfer entropy (TE) estimation, and dynamic causal modeling (DCM). TE and DCM are complementary techniques; TE quantifies information transfer between network nodes, i.e., it focuses on network links that channel new information into a node for computation (Wibral et al., 2011). In terms of inference, TE is an exploratory technique while DCM models the physiological coupling between hidden states of the network nodes (Friston et al., 2013), and is a confirmatory approach based on comparing models. Hence, we used the TE-derived network as the basis to create the family of DCM models to confirm the network structure relevant for the perception of the SiFi through model comparison. In the winning network, we then tested for quantitative variations in connection strength with age, which are indicative of increased influences of cross-modal predictions in older participants.

## 2. Methods

### 2.1. Participants

Twenty five healthy young adults (11 males) between the ages of 21 years to 28 years (mean = 26.96, SD = 2.13 years) and 28 healthy older adults (12 males) between the ages of 58 to 72 (mean = 66.84, SD = 8.15 years) years took part in the experiment. All participants were right-handed. Older adults were given the Consortium to Establish a Registry for Alzheimer disease (CERAD) questionnaire to ensure they did not suffer from age-related cognitive deficits (Morris et al., 1988; Fillenbaum et al., 2008). All participants were also given the d2 attention test (Brickenkamp, 1998) and performed within their age-related norms. All participants gave written informed consent in accordance with the Declaration of Helsinki on biomedical research involving human subjects (Tokyo amendment). The study was approved by the ethics committee of the Goethe University of Frankfurt medical faculty.

### 2.2. Behavioral-Only SiFi

#### 2.2.1. Apparatus and Stimuli

The visual stimuli were presented on a 24 inch flat panel computer monitor with a refresh rate of 60 Hz. The visual stimulus consisted of a white circular disk (diameter 2°), placed 8° of visual angle below the fixation cross. The presentation duration of the disk was 16 ms.

The auditory stimulus consisted of a 16 ms, 3,500 Hz pure tone with a total rise- and decay-time of 20 μs and a sound pressure level at approximately 65 dB A. The auditory stimuli were presented using closed, circum-aural headphones (AKG, Austria, model: K271).

#### 2.2.2. Design and Procedures

The design of the experiment was based on a 3x7 repeated-measures design with factors *Modality* (vision-only, auditory-only, and audiovisual) and *Stimulus-Onset Asynchrony* (SOA; 50, 100, 150, 200, 250, 300, 500 ms). The dependent variable was accuracy. The factor *Modality* was blocked and the order randomized between participants. Participants received instructions and were given a short practice block to ensure they understood the task.

**Figure 1:**
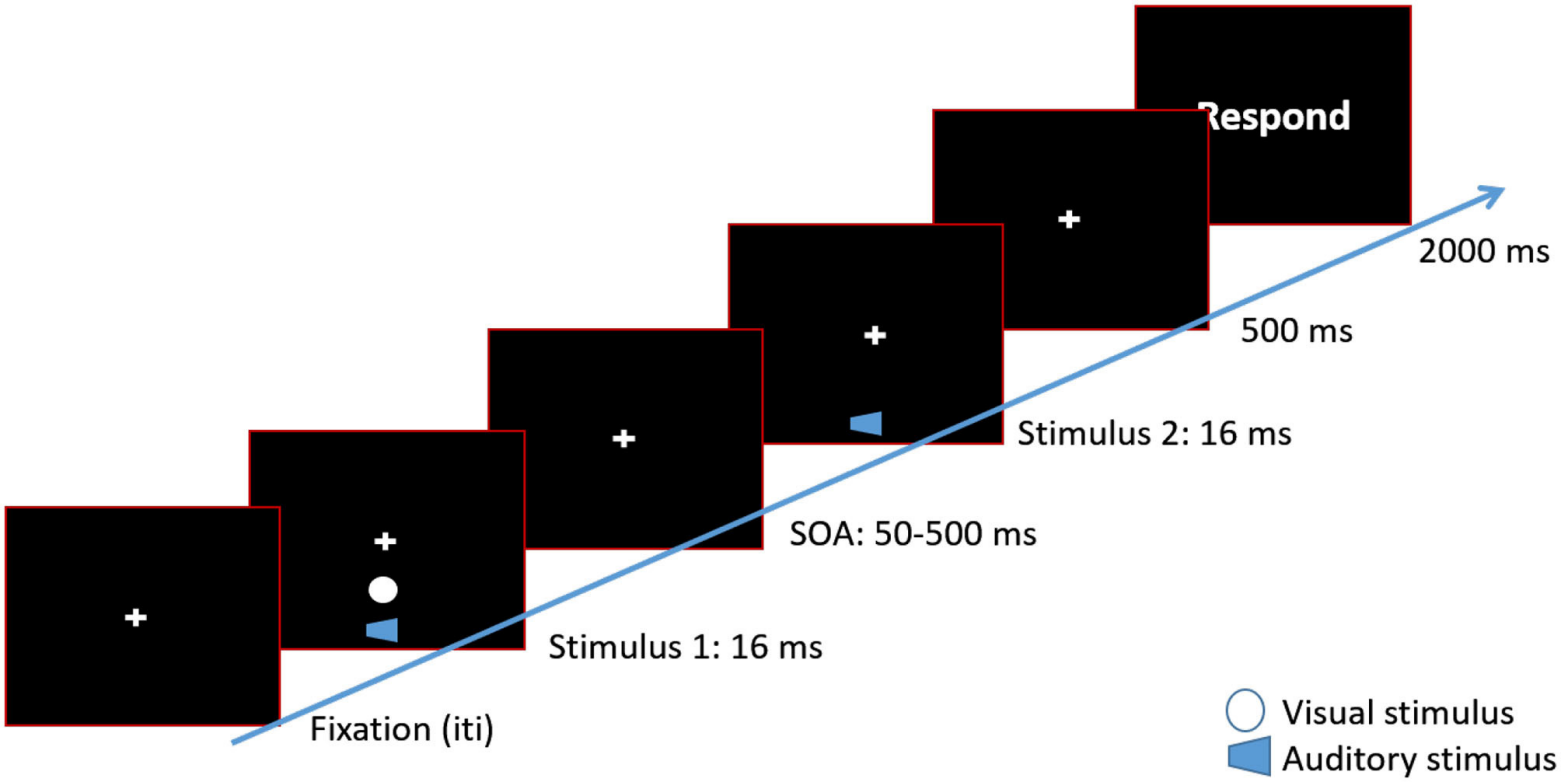
Timeline of a 2 beeps/1 flash condition. Each trial began with a fixation cross at the center of the screen. After a variable inter-trial interval, a white disk (flash) was presented along with a 3,500 Hz tone (beep) for 16 ms. The visual disk was presented approximately 8° of visual angle below the fixation cross. After a variable SOA, the second beep was presented. In the 2 beeps/2 flashes condition a second white disk would be presented along with the second beep. After 500 ms, a response screen was presented. Participants waited for the response screen to make their button press.

Within each block, the participants' task was to indicate how many stimuli (visual or auditory) were presented. At the beginning of each trial, a fixation cross was presented at the center of the computer screen. Participants were instructed to maintain their eye gaze on the cross throughout the experiment. If two stimuli were presented, the first stimulus was presented followed by a variable SOA, between 50 and 500 ms. Then, the second stimulus was presented. Afterwards, participants indicated via button press how many stimuli were presented. In audiovisual blocks, participants were asked to indicate the number of visual stimuli. Each trial was followed by an inter-trial interval (ITI) between 1,000 and 1,500 ms (step sizes of 250 ms; see Figure 1). Participants were asked to emphasize accuracy over speed when indicating the number of stimuli. The experiment was programmed in Presentation (Neurobehavioral Systems, CA, USA).

The unimodal-only conditions (vision-only and auditory-only) were separated into two separate blocks. In the vision-only block, one or two flashes were presented and the participants' task was to indicate how many flashes were presented. In the auditory-only block, one or two beeps were presented and the participant's task was to indicate how many beeps they heard. There were 140 trials in each of the unimodal blocks, 70 trials where one stimulus was presented and the remaining trials where two stimuli were presented, with an equal number divided between the SOA conditions.

The audiovisual block contained two control conditions (1 beep/1 flash and 2 beeps/2 flashes) as well as the illusion condition (2 beeps/1 flash), resulting in 210 trials in total. In the illusion condition, the visual flash was presented at the same time as the first auditory beep. The second beep was presented at one of the previously indicated SOAs. In the control conditions, the audio-visual pairs were presented simultaneously. The second beep-flash pair in the 2 beeps/2 flashes control condition was presented at one of the previously indicated SOA, relative to the first pair. This was done to minimize response bias toward responding to the auditory stimuli. The participants' task was to ignore the auditory stimuli and indicate how many visual flashes were presented. All responses were made using their right hand, via computer keyboard.

### 2.3. MEG Methods

MEG was recorded in accordance to suggested guidelines (Gross et al., 2013). Participants were seated in a 275-channel, whole-head MEG with axial gradients (Omega 2005, VSM MedTech Ltd., BC, Canada). The sampling rate was 1,200 Hz. Data were transformed to a synthetic third order axial gradient representation, and band-pass filtered in hardware between 0.1 and 300 HZ. Four electrooculogram (EOG), two electromyogram (EMG), and two electrocardiogram (ECG) electrodes were placed on the participant's face and clavicles to record eye blinks, facial movements, and heart rate, respectively. Head localization was recorded continuously.

Visual stimuli were delivered via a video projector (Sanyo xp41) and back-projected to a semi-transparent screen at a distance of 60 cm from the participant's head. Auditory stimuli were generated by a computer sound card (Creative Labs; Audigy 32) before going through sound-conducting tubes into the MEG chamber. These sound-conducting tubes were connected to plastic ear molds (ProPlugs, Doc's). The sound pressure level was the same as in the behavioral experiment.

The task inside the MEG was similar to the task outside the scanner, with the following differences. To optimize the number of illusion trials, we used four experimental conditions (2 beeps/1 flash, 2 flashes, 1 flash, and 2 beeps), which were randomly permuted within each block. In the 2 beeps/1 flash, 2 beeps, and 2 flashes conditions, the second stimulus was always presented at a fixed SOA of 100 ms. After 500 ms, the response screen was presented, and participants indicated how many flashes were presented. In the 2 beep condition, participants were instructed to respond “0 flashes.” Trials were rejected if participants responded before the response screen. The inter-trial interval was jittered between 850 and 1,250 ms, in 250 ms steps.

There were 200 repetitions of the 2 beeps/1 flash condition, 100 repetitions of the 1 flash condition, and 100 repetitions of the 2 beeps condition. Fifty repetitions of the 2 flashes conditions were presented additionally to minimize the possible bias to respond “1 flash.” Participants responded using a MEG compatible 5-button response box (Cambridge Research Systems, LTD) with their right hand. All trials were randomized across 5 experimental runs. Each run lasted approximately 7 min. Participants received a short break between each run.

### 2.4. MEG Analyses

#### 2.4.1. Pre-processing

All MEG data processing, except TE and DCM analyses, was carried out using FieldTrip (Oostenveld et al., 2011). Eight sensors (MRF22, MLT44, MRC12, MRC25, MRF22, MRO21, MRO53, and MRF11) had elevated noise levels and were thus excluded from the analyses.

Epochs were cut from 1,000 ms before the onset of the first audio-visual stimulus to 620 ms after the first stimulus. Trials containing eye blinks or muscle artifacts were removed by automated artifact rejection routines and subsequent visual inspection.

On average, young adults perceived 61.80 % of 2 beeps/1 flash trials (122 trials) as illusions, older adults perceived 64.74 % of 2 beeps/1 flash trials (130 trials) as illusion, in the MEG scanner. Three young and twelve older adults were removed from the analyses due to excessive head movement. After artifact rejection, between 50 and 120 trials remained in each condition.

Participants within each age group were rank-ordered based on the proportion of perceived illusions in trials remaining after artifact correction. Then, a median-split was taken to place participants into two groups [Propensity to Perceive Illusion (PPI) and Propensity to Perceive No Illusion (PPNI)]. As a result, after pre-processing, 12 young adults were placed in the PPI group and 10 young adults in the PPNI group, the rate of perceived illusions in the PPNI group was 35 % or less. There were seven older adults placed in the PPI group, and nine older adults in the PPNI group, the rate of perceived illusions in the PPNI group was 24 % or less (mean of perceived illusion in each group: Young PPI = 67.52 %; Young PPNI = 11.57 %; Older PPI = 75.76 %; Older PPNI 10.44 %). Interestingly, the number of trials that survived artifact correction was considerably smaller in the PPI conditions, in older adults, and vice versa for younger adults. It is unclear why this was the case.

To ensure that the MEG results were not confounded by an uneven number of trials between the PPI and PPNI group, trials were stratified such that an equal number of trials was present in both groups.

#### 2.4.2. Time-Frequency Analyses

A time-frequency analysis was computed using Morlet wavelets (wavelet length = 5 cycles, size of the Gaussian taper = 3). This procedure resulted in a single-trial estimation of 2 to 60 Hz power, in 2 Hz steps. The baseline interval was −1,000 to −500 ms before the onset of the first stimulus. Finally, trials for each condition were averaged for each participant.

We extended the cluster-based permutation statistics implemented in the FieldTrip toolbox to a 2x2 independent groups ANOVA, with factors *Age Group* (young vs. older) and *Propensity for Illusion* (low percentage of perceived illusion vs. high percentage of perceived no illusion trials). Induced power for each condition was averaged within each participant, then submitted to an ANOVA, and *F*-values for the main effects or the interaction were computed for each sensor. Sensors where the *F*-value surpassed the critical *F*-value corresponding to an alpha level of 0.05 were selected and assigned to clusters based on their spatial adjacency. Neighboring sensors were defined based on the template-approach implemented in FieldTrip. The average minimum of neighboring channels for the cluster analysis was 8.7 neighbors. Cluster-level statistics were calculated by taking the sum of the *F*-values within each cluster. These calculations were performed for each main effect and the interaction separately. The observed cluster-level statistics were then tested against the distribution of the maximum cluster-level statistics gained from Monte-Carlo simulations with 2,000 permutations for each effect. At each permutation, group and condition assignments were shuffled and the estimation of *F*-values and the clustering procedure were repeated on the resampled data. The resulting maximum cluster values were used to construct the maximum cluster-level distribution under the null hypothesis. Clusters were considered to be significant at an alpha level of 0.05 if the originally observed cluster value was greater than the 95th percentile of the maximum cluster-level statistic distribution. Cluster-based statistical tests effectively circumvent the multiple comparison problem by reducing the dependent variable to the maximum cluster size of neighboring data bins showing the same effect (Maris and Oostenveld, 2007).

Special care must be taken to define the appropriate permutations for a factorial design (Anderson and Braak, 2003; Suckling and Bullmore, 2004). Permutations were restricted to occur within each factor (e.g., *Age Group*), while the assignment of participants to levels of the other factor (e.g., *Propensity for Illusion*) was kept constant. For example, when testing the main effect of *Age Group*, the factor *Propensity for Illusion* was held constant. No exact permutation tests based on the *F*-statistic exist for the interaction effect; since restricting permutation of the observations such that neither group main effect affects the corresponding *F*-ratio would leave no possible permutations of the data. An approximate test was constructed by restricting permutations of factor levels to occur between one factor and subsequently permuting whole subjects across groups. Though variability due to the main effects is not held constant under such a permutation scheme, their variability impinges on all terms of the model, giving a reasonable approximate test (Anderson and Braak, 2003; Suckling and Bullmore, 2004; Haegens et al., 2014; Brodski et al., 2015).

#### 2.4.3. Source Reconstruction

Dynamic imaging of coherent sources (DICS; Groß et al., 2001), a frequency-domain adaptive spatial filtering algorithm in the FieldTrip toolbox, was used to identify the sources of the effects found at the sensor-level. While the DICS algorithm was designed to compute source coherence estimates, here we used only real-valued filter coefficients, and therefore restricted our analysis to the local source power (Grützner et al., 2010). The real-part of the filters reflects the propagation of the magnetic fields from sources to sensors, as this process is supposed to happen instantaneously (Nunez and Srinivasan, 2006). First, illusion and no-illusion trials (i.e., all data) were combined into one data set for each subject. Cross-spectral density matrices were computed for the task period of −250 to 75 ms, in the beta-band, based on the statistical analysis of spectral power at the sensor level (spectral smoothing indicated in parenthesis): 21 Hz (9 Hz). Subsequently, data from both illusion and no-illusion trials were projected separately into source space using the common spatial filter from the previous step. Source analysis was conducted separately on the activity of the two conditions, and the difference between the projected sources was tested for significance as described above. Source activity was interpolated onto individual anatomical images from magnetic resonance imaging (MRI) and subsequently normalized onto the standard Montreal Neurological Institute (MNI) brain using SPM8 in order to calculate group statistics and for illustrative purposes. A linearly constrained minimum variance (LCMV, Van Veen et al., 1997) approach was used to project the frequencies of interest into source space (Blinowska, 2011) to reconstruct source time courses. Common filters were regularized at 5 % (Brookes et al., 2008).

Beamformer filters were computed as “common filters” based on the activation and baseline data across all conditions. Using common filters for activation, baseline, and all conditions allows for subsequent testing for differences between conditions; using common filters ensures that differences in source activity do not reflect differences between filters. Spatial filtering of the sensor data for source statistics was then performed by projecting single trials through the common filter for each condition separately.

#### 2.4.4. Connectivity Analyses

To infer the network underlying the perception of the SiFi, we used a combination of transfer entropy (TE) estimation and dynamic causal modeling (DCM). DCM uses Bayesian inference to obtain the most likely model of physiological interactions given the data. This Bayesian approach requires that plausible models enter the DCM analysis as priors. A common approach that we also followed here, is to define models from relevant neural sources determined by source analyses. Furthermore, we were interested in the interaction between the found sources of activity and the primary auditory (Brodmann area BA22) and visual areas (BA18; Mishra et al., 2007). Previous studies using dynamic causal modeling (DCM) had to balance the number of models that needed to be created from identified sources vs. computational time. This is because if all possible models were to be built from a set of identified sources and connections, this would result in an intractable model space. For example, in the present study, we identified eight neural sources—to explore the entire resulting model space using DCM, would require the generation of 2^28^ = 268, 435, 456 models, i.e., $\frac{1}{2}n\left(n-1\right)=28$ possibilities for each type of model connection (excitatory, inhibitory, and mixed).

This brute-force approach is computationally intractable. Furthermore, the models specified in DCM must be biophysically motivated and may not be randomly generated (Stephan et al., 2010; Friston et al., 2011). Yet, it is rarely the case that all effective connections (or lack thereof) are known between each of the cortical areas in question. To reduce the model space to a tractable size and to plausible models only, we took advantage of the MEG's temporal precision and estimated TE between source time courses. TE is a model-free measure of information transfer between two processes (Schreiber, 2000); the resulting network of information transfer between neural sources represents a candidate connectivity that is relevant for solving a given task. Because we estimated bivariate TE from multiple sources it is very likely that some of the inferred links are indeed spurious due to cascade or common drive effects (Brodski et al., 2015; Wollstadt et al., 2015). The TE network may thus be a highly plausible starting point for building a model of effective connectivity underlying the neural computation but should be further refined using DCM and model comparison.

TE is a model-free measure of information transfer, it quantifies the additional information we can gain about a random process *Y* if we not only know *Y*'s past, but also the past of a second process *X* (Schreiber, 2000). Information transfer is then quantified as the conditional mutual information,

(1) $$\begin{array}{l} TE\left(X\to Y,t,u\right)=I\left(Y_{t};{\text{X}}_{t-u}|{\text{Y}}_{t-1}\right), \end{array}$$

between the future value *Y*_*t*_ of process *Y* at time *t*, and past state **X**_*t*−*u*_, conditional on the past state **Y**_*t*−1_. Here, *u* is the reconstructed physical interaction delay *δ* between both processes. The delay is reconstructed by finding

(2) $$\begin{array}{l} \delta =\underset{u}{arg max}\left(TE\left(X\to Y,t,u\right)\right). \end{array}$$

Prior to TE estimation, we reconstructed states **X**_*t*−*u*_ and **Y**_*t*−1_ from scalar time series using a time-delay embedding (Takens, 1981), with embedding parameters found individually for each participant through optimization of Ragwitz' criterion (Ragwitz and Kantz, 2002; Wibral et al., 2013). Parameter optimization and delay-sensitive TE estimation from the ensemble of trials was done using the MATLAB toolbox TRENTOOL (Lindner et al., 2011; Wollstadt et al., 2014), that implements the Kraskov-Stögbauer-Grassberger estimator for mutual information (Kraskov et al., 2004). We used permutation testing against shuffled surrogate data to establish statistical significance for estimated TE values (Lindner et al., 2011).

We estimated TE for each possible pairwise connection in individual subjects, obtaining single-subject networks of information transfer. From single-subject networks we constructed group-level networks for the PPI and PPNI groups, by including links that were significant in at least 50 % of the subjects within a group. The thresholding procedure corresponds to a one-sided Binomial test over subjects under the null hypothesis of significant links *k* being *B*(*n, p*_0_)-distributed, with *n* = 19 and *p*_0_ = 0.5 for the PPI group and *n* = 15 and *p*_0_ = 0.5 for the PPNI group. The threshold of 50 % significant links is equivalent to an alpha level of 1e−10. We combined both group-level networks by taking the union of both sets of links.

Using the resulting TE network, the model space for DCM was dramatically reduced. DCM was performed using the Statistical Parametric Mapping Matlab toolbox, version 8 (SPM8) (Litvak et al., 2011). To avoid statistical “double-dipping,” TE estimation was performed on the odd-numbered trials and DCM on the remaining even trials for each participant. Overall, three separate DCM analyses were conducted (see below). All DCMs were fit to the remaining (even) trials. As the activation occurred mostly before the presentation of the stimuli, we employed a resting-state paradigm, using the linear neural mass model to calculate cross-spectral density of steady-state responses (Moran et al., 2009; Schmidt et al., 2014). The time window was from 250 ms before stimuli onset to 75 ms post-stimuli onset. The data was detrended by simply removing the mean and the data was not subsampled. Eight modes were selected. Wavelet parameters were the same as those used to calculate the induced activity at the sensor-level.

A single equivalent current dipole for each source was selected as the electromagnetic model. The sources included into the model were the right middle temporal gyrus, right middle frontal gyrus, left fusiform gyrus, right fusiform gyrus, bilateral primary auditory cortex (BA22), and bilateral primary visual cortex (BA18). Network inputs were not selected given that this was a resting-state DCM design. Random effects Bayesian model selection (BMS) was utilized to take into account the inter-individual variability in the structure of each model (Bastos, 2013). Separate model families were created for each type of interaction available (excitatory, inhibitory, and mixed). Then, a separate BMS was performed within the winning interaction-type family. In all cases, the family with purely excitatory interactions was the overall winner. Subsequently, the individual models within the excitatory family were compared using Bayesian Model Selection (Penny et al., 2004; Kiebel et al., 2008).

For all three DCM analysis steps, the union of the PPI and PPNI TE networks was the basis for all investigated DCM models. A hierarchical approach was taken for the analyses, where the first two steps tested for spurious links in the TE network, to obtain a parsimonious common model, and the third step tested for age-related modulations in the winning model. To determine the winning model, we used the model comparison statistics in the Variational Bayesian Analyses toolbox (Daunizeau et al., 2014). In the first step, we tested the full model, comprising all links and all links were modulated. Also we tested all models created through the removal of a single link from the full model, where all remaining links were modulated. This step aimed at identifying potentially spurious links by testing of whether removing a link increased the model evidence. The frequency range of interest for DCM was limited to the beta-band (12 to 25 Hz), because the network nodes had been defined via the sources found in this band.

In the second DCM analysis step, triggered by the finding that pruning links increases model evidence, we tested the winning model from analysis step one for simple common drive and cascade effects by removing multiple potentially spurious network links simultaneously from the winning model from the first DCM step (Kamiński et al., 2001; Blinowska et al., 2004; Vakorin et al., 2009). This had to be done in a systematic and tractable fashion as scanning models with all further possible combinations would have resulted in a set of computationally prohibitive size. We therefore employed a strategy where pruned links constrained each other based on membership in acyclic triangles in the network graph. Acyclic triangles may indicate spurious links, either due to one node driving the dynamics in the other two nodes (common drive effect), or due to a cascade of information transfer, where two consecutive links lead to spurious information transfer between the first and the third node (cascade effect; Wollstadt et al., 2015). In such an acyclic triangle, only one of the two potentially spurious links can be actually spurious, because the two effects are mutually exclusive. This leads to a set of constraints on possible link-removals when trying to account for cascade and common driver effects.

Thus, in the alternative models, we systematically destroyed acyclic triangles, while making sure that no more than one of the two potentially spurious links was removed from all triangles in any given model. Possible combinations of simultaneously removable links were identified by encoding removable links as a Boolean function.

After estimating model evidences for all candidate models, we tested the hypothesis that the model distributions differed between age groups vs. the hypothesis that it was not different. To this end we used Bayesian group comparison as implemented in the Variational Bayesian Analyses toolbox (Daunizeau et al., 2014). Accordingly, the third step of the DCM analysis was carried out on the common winning model for both groups.

A third DCM analysis was conducted on the winning model from the second (refined) DCM analysis, to determine which links were modulated by illusory percepts. In this analysis, all links were maintained (A-matrix) but the modulation of individual links was systematically removed (B-matrix). We then statistically compared the illusion-trial related modulation in connectivity strength between the young and the older age group using *t*-tests.

## 3. Results

### 3.1. Increased SiFi in Older Participants

In order to determine the earliest illusion stimulus-onset asynchrony (SOA) which differentiates the age groups, participants were presented with a behavioral version of the SiFi to assess their temporal binding window outside the MEG. In the illusion condition, two beeps and one flash were presented (2 beeps/1 flash). The SOA between the auditory stimuli varied between 50, 100, 150, 200, 250, 300, and 500 ms. Control conditions were also presented (2 beeps/2 flashes and 1 beep/1 flash), with control and 2 beeps/1 flash trials randomly permuted within a single block. Participants indicated the number of perceived flashes. In additional unimodal conditions, presented in separate blocks, participants indicated the number of beeps or flashes (see Supplementary Material).

**Figure 2:**
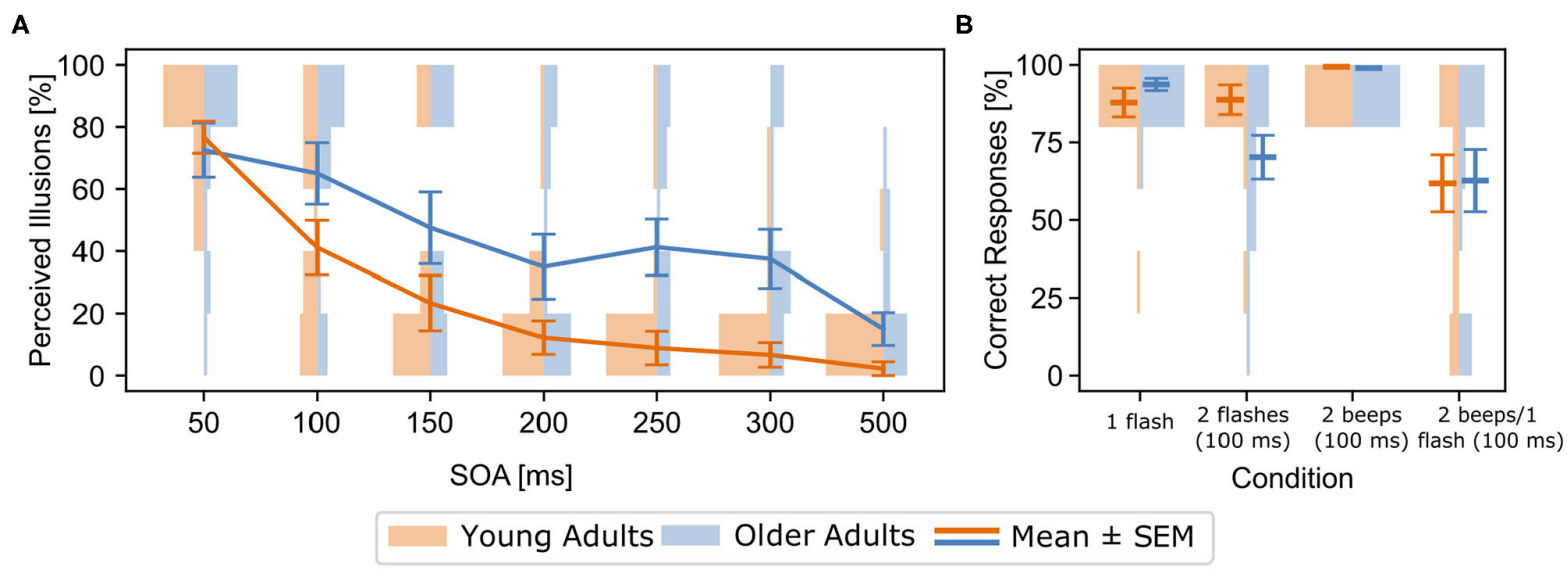
**(A)** [Behavioral results for the 2 beeps/1 flash condition (means and standard errors (SEM))] Rates of perceived illusions in the experimental setup outside the MEG for all stimulus-onset asynchronies (SOA) and both age groups (blue: older adults, orange: young adults). Line graphs show means and standard errors (SEM), histograms show distribution of rates for each group and SOA. Rates denote incorrect responses (i.e., perceived illusions) in the 2 beeps/1 flash condition. Older adults perceived more illusions across the 100-500 ms SOA conditions, compared to young adults. **(B)** Behavioral results from within the MEG for both groups and all experimental conditions. Line graphs show means and SEM, histograms show distribution of rates for each group and condition. Rates denote correct responses for each condition. Note that incorrect responses in the 2 beeps/1 flash condition indicate the perception of an illusions.

Older adults perceived significantly more illusions than young adults [*F*_(1, 34)_= 6.31, $\eta_{p}^{2}=0.15$, *p* = 0.02, Figure 2A]. There was also a significant *Age Group x SOA* interaction in the 2 beeps/1 flash condition [*F*_(6, 204)_ = 3.19, $\eta_{p}^{2}=0.09$, *p* = 0.005], driven by more perceived illusions for older compared to younger adults between SOAs 100 and 500 ms, but not at a SOA of 50 ms (see Figure 2A). Illusion perception between the age groups began to diverge at 100 ms (*p* = 0.002). These effects were not related to response bias, as there were no group differences in the multisensory control conditions (see Supplementary Material, also for unimodal results).

### 3.2. Neurophysiological Signature of the Illusory Perception

In order to determine the neural activity underlying the increased illusion perception, both groups performed the same task inside the MEG—with the exception that only the 100 ms SOA: 2 beeps/1 flash condition, 2 beeps only, 1 flash only, and 2 flash only conditions were presented. This was done to optimize the number of illusion trials.

We performed a sensor-based 2x2 mixed-design cluster permutation ANOVA with *Age Group* (young vs. older) as the between-subjects factor and *Propensity for Illusion* (trials where participants perceived the illusion vs. trials where participants did not perceive the illusion) within the beta-band (12 to 25 Hz). There was a significant main effect of *Age Group* (-0.5 s–0.55 ms; *p* = 0.007), with older adults exhibiting greater beta-band activity compared to younger adults. There was no main effect of *Propensity for Illusion* (*p* = 0.47) or significant interaction (*p* = 0.40).

On average, the sound-induced flash illusion occurred in about 60 to 70% of trials, with some participants showing a greater propensity to perceive the illusion compared to others. To understand whether an age-independent factor determined a subject's propensity for perceiving an illusion, we performed a 2x2 between-groups permutation-based ANOVA with factors *Age Group* (young vs. older) and *Propensity for Illusions* [perceived illusion (PPI) vs. perceived no illusion (PPNI)] on the squared amplitude of the sensor-level time-frequency transformed data. Older adults had significantly greater beta-band activity (12 to 30 Hz) compared to young adults in the time range of −250 to 75 ms relative to the onset of the first stimulus (*p* = 0.002). However, there was no main effect of the factor *Propensity for Illusions* nor an interaction between both factors. There were no significant differences in other frequency bands (i.e., theta-, alpha-, or gamma-bands).

**Figure 3:**
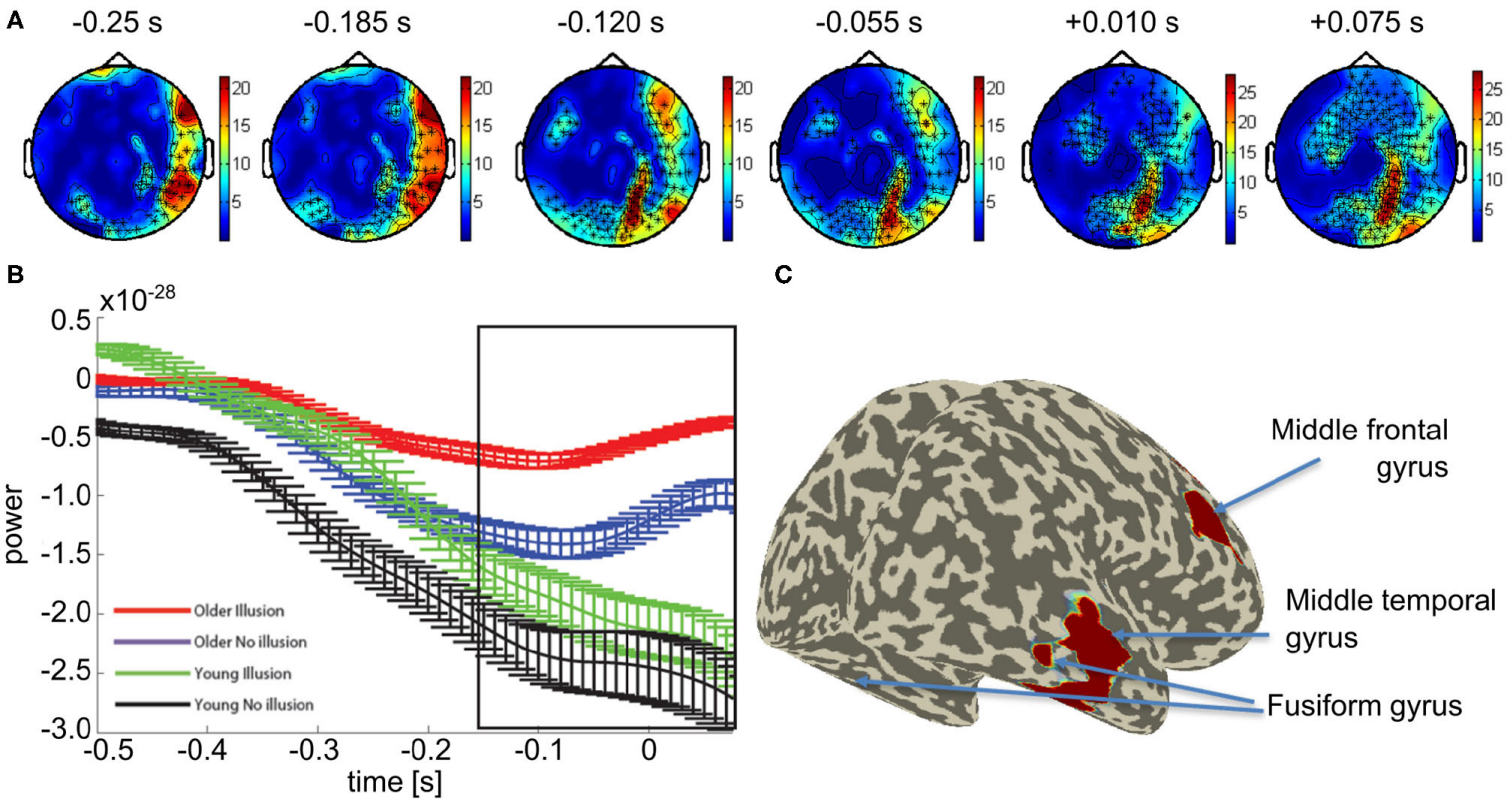
Mean effect of *age group* in the beta band: **(A)** Topological plot showing locations of the significant MEG channels (black crosses). **(B)** Average beta-band power over time for significant channels (means and SEM, see Supplementary Figures 4–7 for non-averaged results). **(C)** Source localization results of the beamformer analysis of beta band power within the time window indicated by the black box in **(B)**.

We focused our further network analysis of the MEG data on the time interval and frequency range of the differential sensor-level statistics between the groups with a propensity to see the illusion vs. those with a propensity to see no illusion in order to determine the network used between groups. First, DICS was used to identify the sources of the increased beta-band activity, across all participants. Beamforming revealed peak activity within the time interval of −250 to 75 ms to occur in the right middle temporal gyrus (Talairach coordinates: 50 -30 -10), right middle frontal gyrus (30 -20 -30), and bilateral fusiform gyrus (±20 -70 -10) (see Figure 3).

### 3.3. Information Transfer Within the SiFi Network

**Figure 4:**
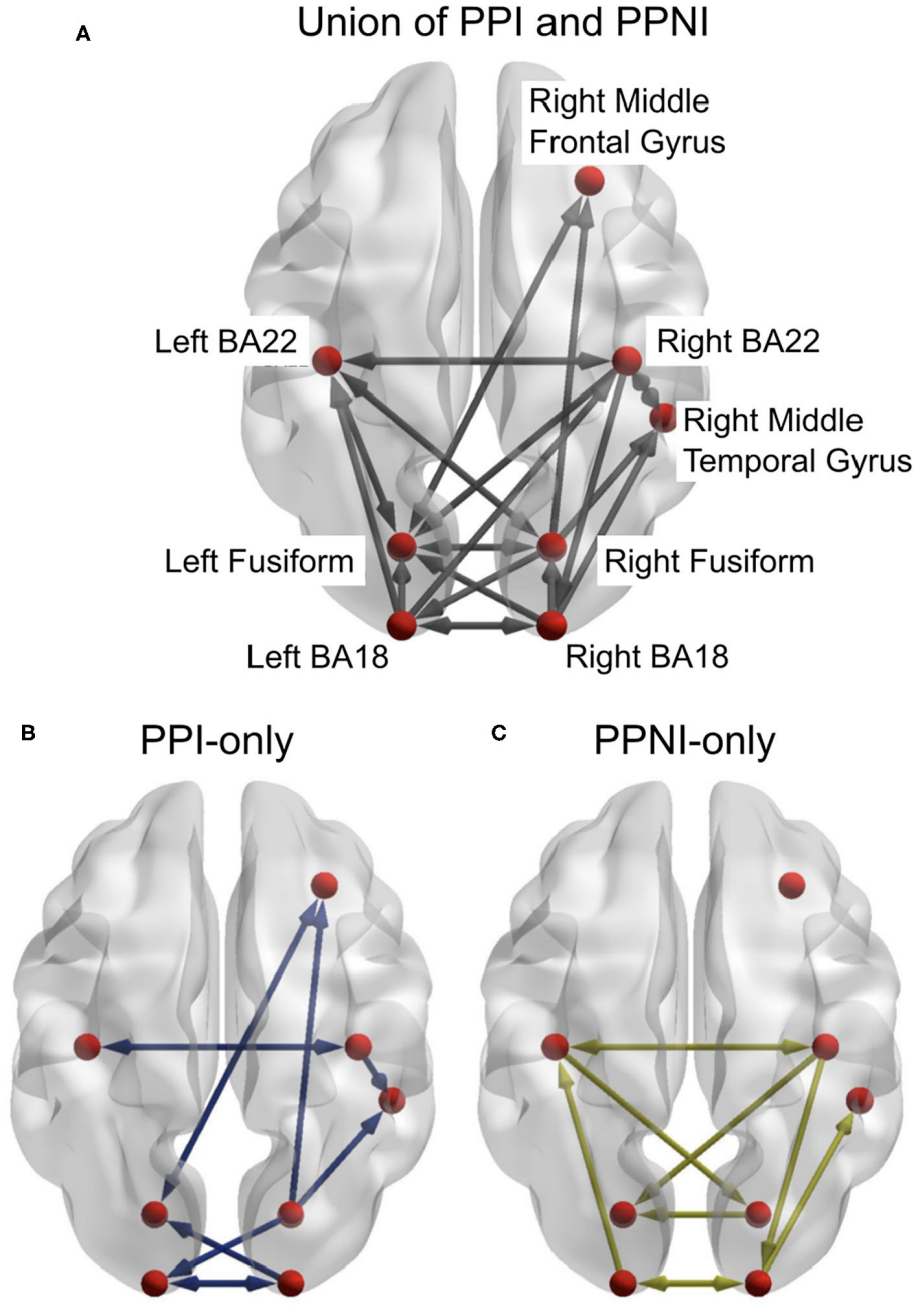
**(A)** The combined results from the transfer entropy analyses of the trials for propensity to perceive illusion (PPI) and propensity to perceive no illusion (PPNI). This also includes links that are associated with both percepts. **(B,C)** represent the differences between the PPI and PPNI group networks, respectively, and the union network. Connections which are present in **(A)** but not in **(B)** or **(C)** indicate that those connections are present in both conditions. Figures generated with BrainNet Viewer (Xia et al., 2013).

We estimated delay-sensitive TE (Wibral et al., 2013) from reconstructed state spaces (Takens, 1981; Ragwitz and Kantz, 2002). We estimated TE for each pairwise combination of sources in each participant and tested these TE values for statistical significance using a permutation test against surrogate data (Lindner et al., 2011). We thus obtained networks for individual participants, which we then combined into group-level networks for propensity to perceived illusion (PPI) and propensity to perceived no illusion (PPNI) across age groups, using a binomial test of individual links across all participants, irrespective of age (see Figure 4).

We then took the union of the group-level networks of the PPI and the PPNI groups and constructed DCM models to find the links that explained differences in performance between the age groups. While the SiFi is a robust illusion, the proportion of perceived illusions is not consistent across individuals within an age group (McGovern et al., 2014). In order to confirm and refine the model, we used a systematic approach, where DCM was applied hierarchically in three steps: the first two steps were aimed at obtaining a parsimonious, common model describing the data for both groups of old and young participants, while the third step then investigated age-related modulations of model parameters in this common model.

The aim of the first DCM analysis step was to determine if the union model offered a good description of the data for both age groups and whether small variations to it would yield higher model evidence, indicating the need for a more thorough pruning of the union network. In this first TE inspired DCM analysis, twenty-four models were generated and applied to both age groups (see Supplementary Figures 8, 9). The links for the DCM models were a union of the PPI and PPNI TE models. The frequency range of interest was constrained to the beta-band. Model 1 consisted of all links, with all links being present (Supplementary Figure 8). Models 2 to 24 systematically removed one link, to identify any possible links whose removal might affect the model evidence (Supplementary Figures 8, 9). Models 25 and 26 (Supplementary Figure 9) consisted of only the links in either the PPI or PPNI TE models, respectively (see Figure 4). The resulting winning model was model 5, which was close to the union model, but had link Right BA18 to Right MTG removed.

**Figure 5:**
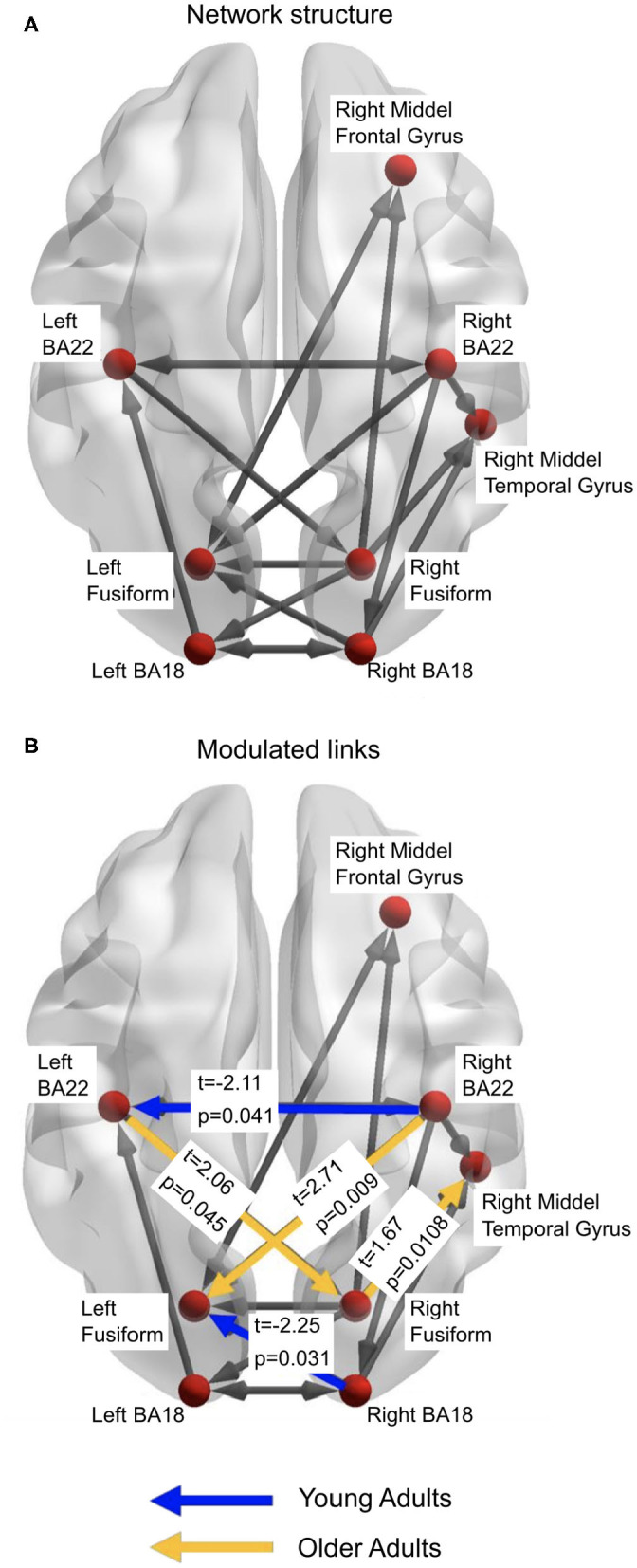
**(A)** The winning model from the DCM analyses. This represents the structure of the network that is active in both young and older adults. **(B)** represents the links which demonstrate significantly more beta-band activity between the age groups. Figures generated with BrainNet Viewer (Xia et al., 2013).

As the first DCM analysis step indicated an improvement of model evidence via pruning of links, the second DCM analysis was conducted to remove additional spurious effects due to common drives and cascade effects from the winning model (5, Supplementary Figure 8) of the previous DCM analysis. This was done based on a link's membership in an acyclic triangle in the directed network graph, as the brute-force approach would have required testing of 227 models. The second analysis step resulted in a set of only 20 models (see Methods and Supplementary Figures 10, 11). The winning model for both young and older adults was model 10 (Supplementary Figure 10) suggesting that the same network is used in both groups. We verified this by comparing the model distributions in the two groups via the model comparison statistics in the Variational Bayesian Analyses toolbox (Daunizeau et al., 2014). There was positive evidence against different model distributions between the two age groups (log Bayes factor = 6.68), confirming that both groups indeed employed the same neural network when perceiving the illusion (see Figure 5A).

The third stage of DCM analyses studied which links in the common network were modulated by the illusory percept in each age group. Thus, we examined the individual modulatory links in the binary connectivity matrix (B-matrix). The results from this DCM analysis found the winning model, with respect to a modulation of connection strength by the illusory percept for young adults (Figure 5A). Separate independent t-tests with factor *Age Group* were conducted for each modulation (see Figure 5B). Older adults showed increased modulation of links: from the left primary auditory (BA22) cortex to right fusiform cortex (*t* = 2.06, *p* = 0.045); right fusiform cortex to right middle temporal gyrus (*t* = 1.67, *p* = 0.011); and right auditory cortex (BA22) to left fusiform cortex (*t* = 2.71, *p* = 0.009), compared to the younger adults. Younger adults had greater modulation of the link from the right visual cortex (BA18) to the left fusiform cortex (*t* = −2.25, *p* = 0.03), and from right BA22 to left BA22 (*t* = −2.11, *p* = 0.041).

## 4. Discussion

Previous studies have demonstrated that older adults as well as some patient populations have increased rates of illusory percepts, which can be interpreted as increased rates of multisensory integration. Predictive coding theories offer a parsimonious explanation for this effect, considering that the amount of accumulated prior information increases over the life-span, while the precision of unisensory evidence decreases. Together these changes should favor interpretations of the world based on priors over veridical ones. Thus, comparing the neural basis of illusory percepts in young and old participants offers both, a critical test of microcircuit theories of predictive coding, as well as an opportunity for novel insights into perceptual changes in aging.

We found that modulations of effective connectivity linked to illusory vs. veridical percepts carried clear signatures of cross-modal predictions in the older participants, whereas modulations in the younger participants were more linked to changes in connections within unisensory networks. This pattern of results aligns with a predictive coding account of aging-related illusory perception. The link between the cross-modal use of priors and illusory percepts in older participants is further strengthened by the observation that older participants had higher activity in the beta-band in the pre-stimulus phase than younger participants. This strengthens the link because recent microcircuit theories of predictive coding suggest that priors or predictions are generated in cortical layers 5 and 6 and are signaled in the beta-band (Bastos et al., 2015). A result seemingly conflicting with the link between beta-band activity and priors (predictions) related to illusions is the fact that we found no significant effect of illusion-propensity across subjects on beta-band activity. Yet, when analyzing illusion vs. no-illusion conditions within subjects, we found a trend toward increased pre-stimulus beta-band activity for trials where an illusion is perceived compared to trials were no illusion is perceived (Supplementary Material). Thus, we attribute the failure to observe beta-band effects of illusion-propensity to the difficulty to separate age and illusion-related effects. A similar effect was found previously (Keil et al., 2014), however, a direct replication was not possible in a later study (Kaiser et al., 2019). Also, the lack of a significant statistical effect is likely due to the heterogeneity of the sample, more specifically the sample of older adults. A difference between perceived illusion and no illusion was found in young adults, which was, however, not large enough to overcome the total model variability when also considering the older adults. It is also important to note that the sample of young adults was relatively small compared to, e.g., Kaiser et al. (2019) and significantly fewer trials were presented compared to Keil et al. (2014). Fewer trials were presented here to minimize fatigue in the older adults.

In addition to the more frequent occurrence of illusory precepts in older participants, they also exhibited more illusory percepts over a wider range of SOA. We interpret this as a widening of the acceptance window for binding auditory and visual events, brought about by the necessity to interpret degraded inputs from the visual system (Setti et al., 2011, 2014).

We note that the illusion-related changes in effective connectivity as detected by our combined information-theoretic and DCM analysis were found between early and late sensory processing areas. This is in contrast to previous research in young adults only, which suggested that illusory percepts were caused by enhanced early sensory integration only (Shams et al., 2005; Mishra et al., 2007; Bolognini et al., 2013). However, it is important to note that beta-band modulations in young adults were primarily between early sensory areas. There are at least three reasons for this discrepancy between these findings. First, mechanisms related to cross-modal illusions may be more detectable in the older participant group as they are more dominant there, favoring detection in our study cohort. Second, transfer entropy compares favorably with other methods of finding connectivity (Lungarella et al., 2007; Vicente et al., 2011) as it is sensitive also to non-linear coupling (e.g., as required by communication between frequency bands). Third, the approach of model comparison based on DCM offers the possibility to disentangle effects due to structural differences in task-related networks (which were absent between groups) and illusion-related modulations of coupling strength (which were present and differed between groups).

Previous studies have suggested an alternative explanation for illusory percepts in older people by relating them to an age-related delay in neural processing (Andrés et al., 2006; Gazzaley et al., 2008; Wascher et al., 2012). This is an unlikely explanation for our findings. The time course of neural activity at the source-level showed that beta-band activity in older adults was not delayed compared to younger adults (see Figure 3B). In fact, in older adults who are more likely to perceive the illusion, the increase in amplitude of the beta-band activity begins slightly earlier than in their younger counterparts. Therefore, the results illustrated here are most likely not due to neural delays caused by aging.

From a methodological perspective, the current study is one of the first to combine exploratory (TE) and confirmatory (DCM) approaches to the analysis of network activity. While this has been suggested theoretically before for the combination of Granger Causality and DCM (Friston et al., 2013), we would like to add some comments related to the practical application. First, we stress that applying the two analyses to separate data sets (e.g., odd and even numbered trials) is necessary to avoid variants of double dipping. Second, after deriving an estimate of the network structure from an exploratory approach, a confirmation of the network structure by model comparison requires the formulation of multiple plausible network models. The original suggestion has been to use an increasing number of eigenmodes of the functional connectivity matrix for this purpose (Seghier and Friston, 2013). Here we opted for a targeted removal of links from triangular network motifs indicative of common driver and cascade effects. This was done because such spurious links are known to appear in bivariate network analyses via transfer entropy, and were considered to be the main obstacle for the a priori validity of our models (Kamiński et al., 2001; Blinowska et al., 2004; Lizier and Rubinov, 2013; Wollstadt et al., 2014).

In sum, our results suggest that the decrease of unisensory acuity and the accumulation of prior knowledge over the life span leads to a perception of the world increasingly dominated by this prior knowledge. Accordingly, older compared to younger adults had increased rates of illusory percepts and showed modulations of cross-modal connections linked to these illusions. At the level of oscillatory neural activity, both aging and the behavioral occurrence of illusions were linked to increases in beta-band activity. This supports recent neurophysiological accounts of predictive coding where priors and predictions are carried by beta-band activity.

## Data Availability Statement

The raw data supporting the conclusions of this article will be made available by the authors, without undue reservation.

## Ethics Statement

The studies involving human participants were reviewed and approved by Ethics committee of the Goethe University of Frankfurt medical faculty. The patients/participants provided their written informed consent to participate in this study.

## Author Contributions

JC designed the experiment and data analysis, carried out the experiment and data analyses, and wrote the manuscript. MW designed the data analysis and wrote the manuscript. CS assisted in carrying out the experiment and performed the DCM analysis. MB assisted in carrying out the experiment. SH conceived of and carried out the permutation ANOVA analysis. MN assisted in designing the experiment. JK designed the experiment and assisted in writing the manuscript. PW carried out the transfer entropy analysis and assisted in writing the manuscript. All authors contributed to the article and approved the submitted version.

## Conflict of Interest

The authors declare that the research was conducted in the absence of any commercial or financial relationships that could be construed as a potential conflict of interest.
