# Supplementary material for: Predictive Coding Over the Lifespan: Increased Reliance on Perceptual Priors in Older Adults—A Magnetoencephalography and Dynamic Causal Modeling Study
Source: Front Aging Neurosci. 2021 Apr 9;13:631599. doi: 10.3389/fnagi.2021.631599 (PMC8062739; doi:10.3389/fnagi.2021.631599)
Supplement: Supplementary file 1 [file Data_Sheet_1.PDF]

Supplementary Material

1 METHODS

1.1 MRI Scanning Parameters

MRI images were collected using a 3 Tesla Siemens Allegra scanner (Siemens, Erlangen, Germany) at the Brain Imaging Center, Frankfurt using a 4 channel head coil. The subjects' position was head-first supine and slice order was descending. A magnetization-prepared rapid acquisition gradient-echo (MP-RAGE) sequence was applied with the following parameters: TR: 2200 ms; TE: 3.93; flip angle: 9°; matrix: 256x256; FOV: 192 mm; voxel size: 1.0x1.0x1.0 mm3; number of slices: 160; distance factor: 50 %. The duration of the sequence was 4 min. These scans were taken several weeks following the MEG testing.

2 RESULTS

2.1 Behavioral

2.1.1 Unimodal

To determine the unimodal temporal acuity for the visual and auditory system alone, 2x2x8 Greenhouse-Geisser corrected mixed ANOVA was conducted with the factors Age Group (young vs. older) as the between-subjects factor, Modality (vision-only vs. auditory-only), and SOA (0 ms, 50 ms, 100 ms, 150 ms, 200 ms, 250 ms, 300 ms, and 500 ms) as the within-subjects factor. There was no main effect of Age Group ($F(1, 25) < 1, n.s.$). There was a main effect of Modality, with greater accuracy in the auditory-only condition (96.96 %) compared to the vision-only condition (87.53 %, $F(1, 25) = 26.82$, $p < 0.0001$). There was also a main effect of SOA, with greater accuracy as the SOA increased (see Figure S1, $F(7, 175) = 26.73$, $p < 0.0001$). There was a significant interaction between Modality and SOA ($F(7, 175) = 8.53$, $p < 0.0001$). A post-hoc analyses revealed that performance in the visual condition was significantly worse than the auditory condition in the 50 ms and 100 ms conditions (for all, $p = 0.05$). Performance in the 50 ms SOA condition was significantly worse than all other SOAs in the vision-only condition (for all, $p = 0.05$). There were no other significant interactions.

2.1.2 Multisensory

To test for statistically relevant differences in accuracy, we conducted A 2x3x7 Greenhouse- Geisser corrected mixed-measures ANOVA was conducted with *Age Group* (young vs. older) as the between-subjects factor and *Modality* (2 flashes vs. 2 beeps vs. 2 beeps/1 flash) and SOA (50 ms, 100 ms, 150 ms, 200 ms, 250 ms, 300 ms, and 500 ms) as within-subjects factors.

There was a significant main effect between young (87.87 %) and older adults (81.60 %, $F(1, 34) = 4.18$, $p = 0.05$). There was a significant main effect between the AV Conditions ($F(2, 68) = 54.10$, $p < 0.0001$), with the highest accuracy in the 1 flash/1 beep (95.12 %) and 2 beeps/2 flashes (95.63 %) compared to the 2 beeps/1 flash condition (63.99 %; all $p < 0.0001$). There was a significant main effect of SOA ($F(6, 204) = 36.60$, $p < 0.0001$), with accuracy improving as the SOA increased ($50ms = 71.24\%$; $100ms = 78.46\%$; $150ms = 84.98\%$; $200ms = 88.21\%$; $250ms = 87.99\%$; $300ms = 88.89\%$; $500ms = 94.63\%$). There was a significant interaction between AV Condition and Age Group ($F(2, 68) = 5.36$, $p = 0.007$). A posthoc test revealed older adults were significantly worse in the 2 beeps/1 flash condition compared to other conditions and young adults, including performance from young adults in the 1 beep/1 flash condition (all $p < 0.01$). There was also a significant difference between young adults in the 2 beeps/1 flash compared to all other conditions (all $p < 0.01$). Finally, there was a significant interaction between the three factors ($F(12, 408) = 3.20$, $p = 0.0002$). Another post hoc revealed performance in the 50 ms 2 beeps/1 flash

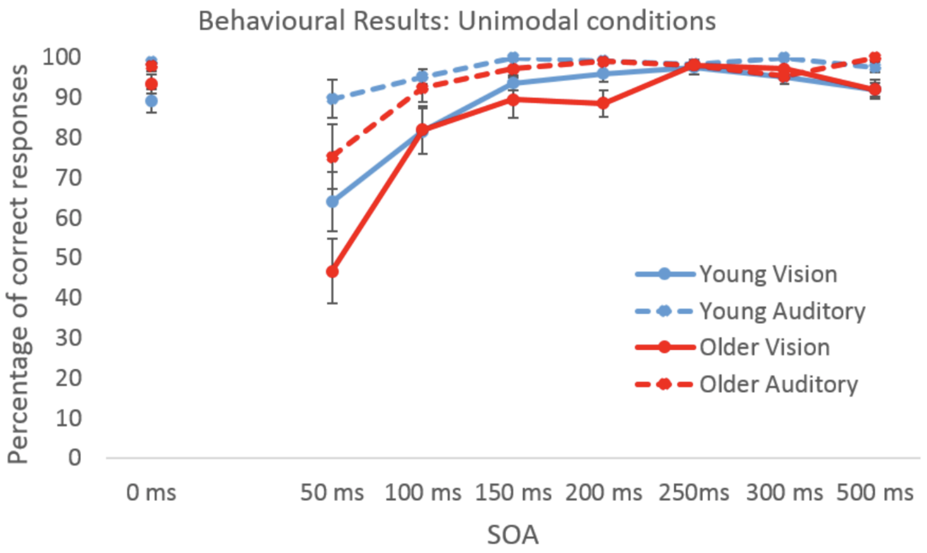

Figure S1. Behavioral performance in the vision-only and auditory-only conditions. Overall, performance was better in the auditory-only conditions compared to the visual-only conditions. There was not significant difference between age groups.

condition was significantly worse when the older and younger participants (all $p < 0.05$; see Figure S2). Older adults perceived more illusions compared to the younger adults, from 100 ms to 300 ms (all $p < 0.05$; see Figure S2). There were no significant differences between the two groups in the control conditions. Furthermore, there was no significant difference between the two groups in either unimodal conditions. The increase in perceived illusion for older adults was not related to a difference in unisensory acuity.

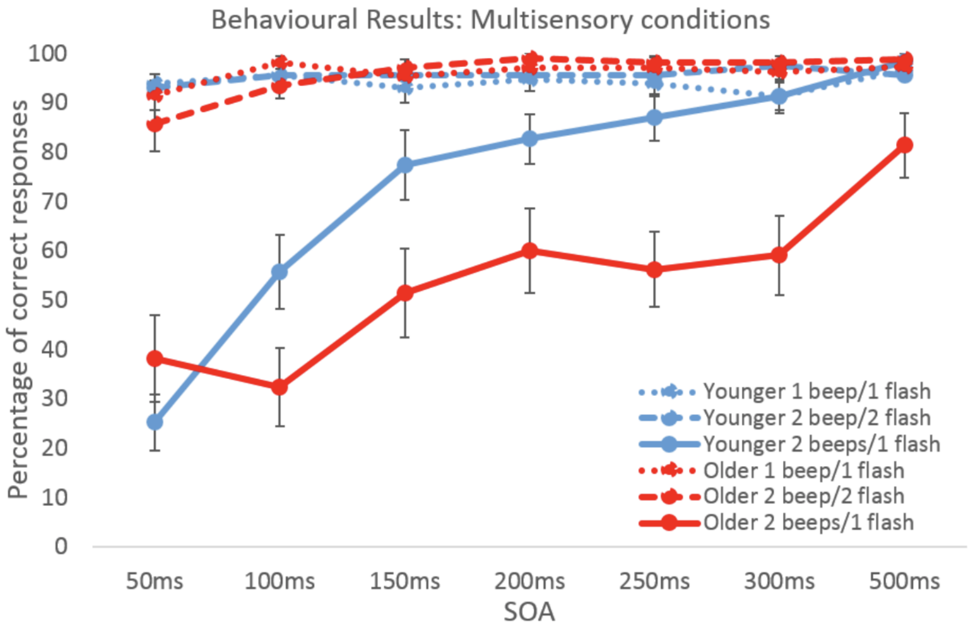

Figure S2. Behavioral performance in the multisensory conditions. There were no significant differences in the two control conditions (2 beeps/2 flashes and 1 beep/1 flash) between the age groups.

2.2 MEG Results

A cluster-based permutation statistics, implemented in the Fieldtrip toolbox, to a 2x3 mixed- design ANOVA with Age Group (young vs. older) and the Illusion (illusion and no-illusion conditions) as the within participants sensor-level analysis revealed a trend towards increased β-band activity to trials where participants (regardless of age) perceived the 2 beeps/1 flash condition as an illusion, compared to no illusion ($p = 0.100$; see Figure S3). This trend occurred between –50 ms to 90 ms over the frontal sensors then extending over the right parietal sensors.

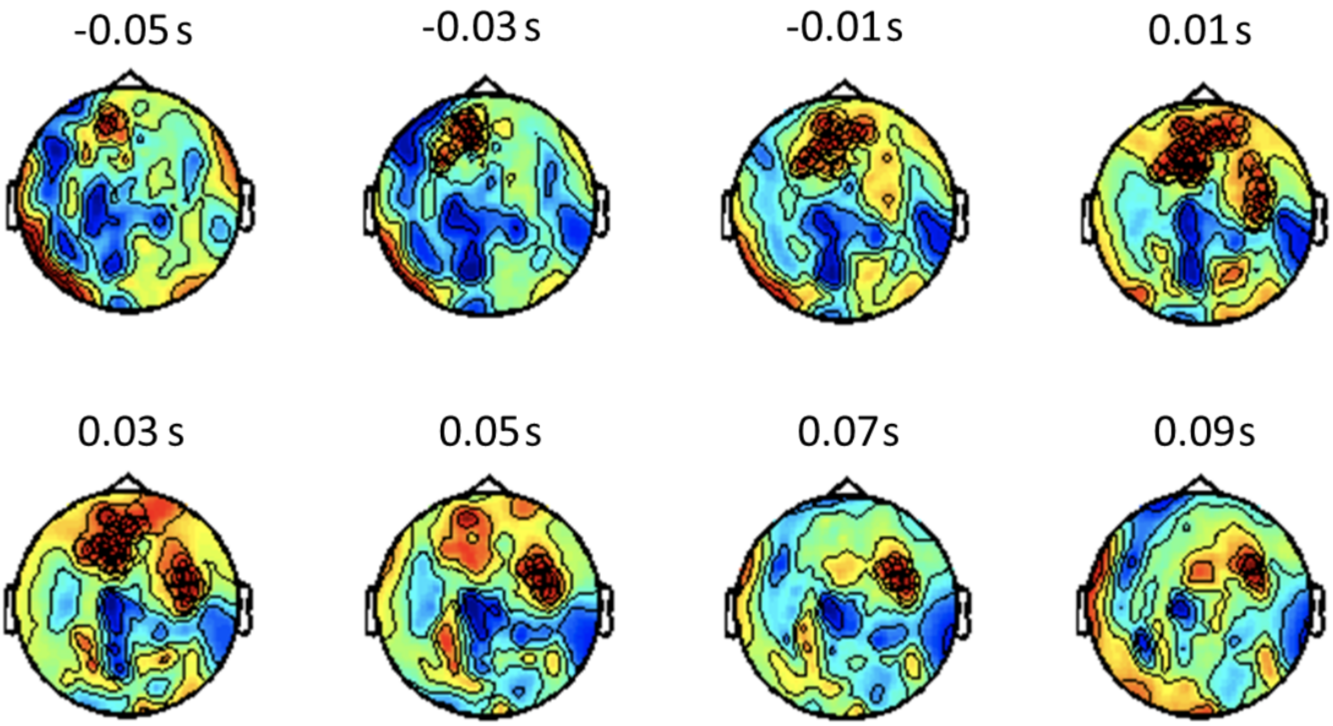

Figure S3. MEG sensors associated with increased β-band activity in perceived illusion trials compared to perceived no-illusion trials. The sensors most associated with the increase in β-band activity are indicated by a black marker.

Figures S4 to S7 show non-averaged power in the β-band for individual sensors over the time interval –500 ms to 620 ms as time-frequency plots. Plots show power over time for young adults in illusion (Figure S4) and no-illusion trials (Figure S5), as well as for older adults for illusion (Figure S6) and no-illusion trials S7.

2.3 DCM results

The winning model for both the young and older adults was model 10 (Figure S7) suggesting that the same network is used for both groups. To compare the Bayesian model Selection between the two groups, we used the model comparison statistics in the Variational Bayesian Analyses toolbox (Daunizeau et al., 2014). There was little model evidence for a different pattern of model evidence between each age group (model evidence $= 6.6789$).

2.3.1 B0 results

The results from the third DCM analysis found the winning model for the young adults was model 9 while the winning model for the older adults was model 7 (Figure S7). Separate independent t-tests were

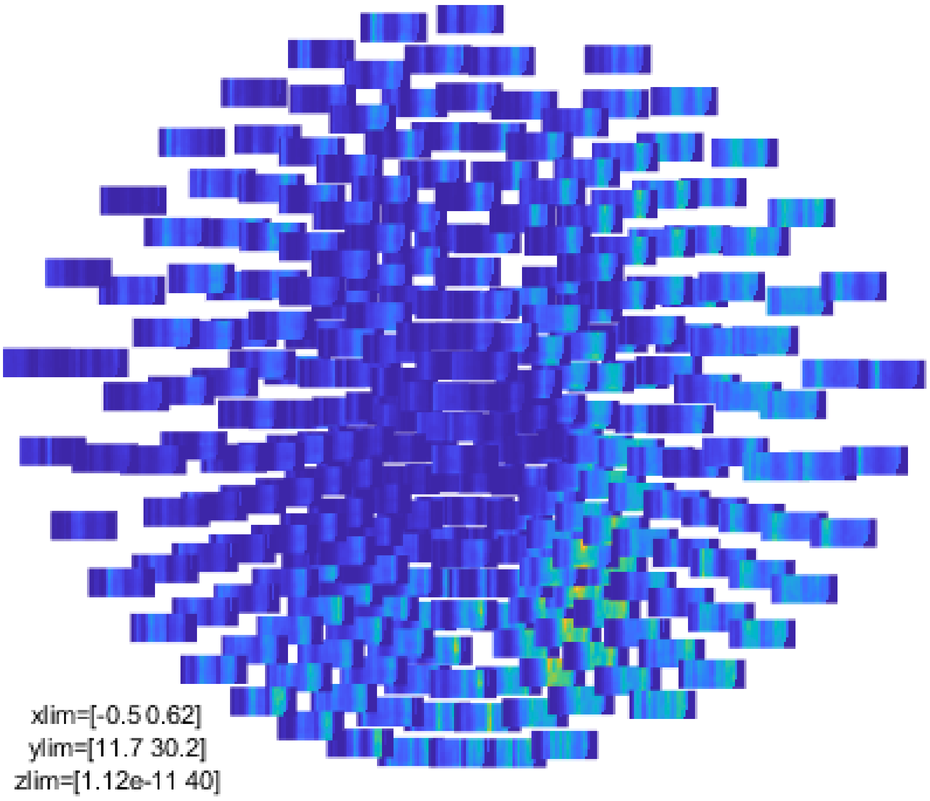

Figure S4. Time-frequency plots for individual MEG sensors for younger adults and trials with perceived illusion. The x-axis denotes time from −500 ms to 620 ms, the y-axis denotes frequency, limited to the β-band (11.7 Hz to 30.2 Hz).

conducted for each modulation (see Figure S8 for a summary of results). Older adults showed increased modulation of the left primary auditory (BA22) cortex to right fusiform cortex ($t = 2.06$, $p = 0.045$); right fusiform cortex to right middle temporal gyrus ($t = 1.67$, $p = 0.011$); and right auditory cortex (BA22) to left fusiform cortex ($t = 2.71$, $p = 0.009$), compared to the younger adults. Younger adults had greater modulation from the right visual cortex (BA18) to the left fusiform cortex ($t = -2.25$, $p = 0.03$), and from right BA22 to left BA22 ($t = -2.11$, $p = 0.041$).

2.3.2 DCM models

REFERENCES

Daunizeau, J., Adam, V., and Rigoux, L. (2014). VBA: a probabilistic treatment of nonlinear models for neurobiological and behavioural data. *PLoS Computational Biology* 10, e1003441

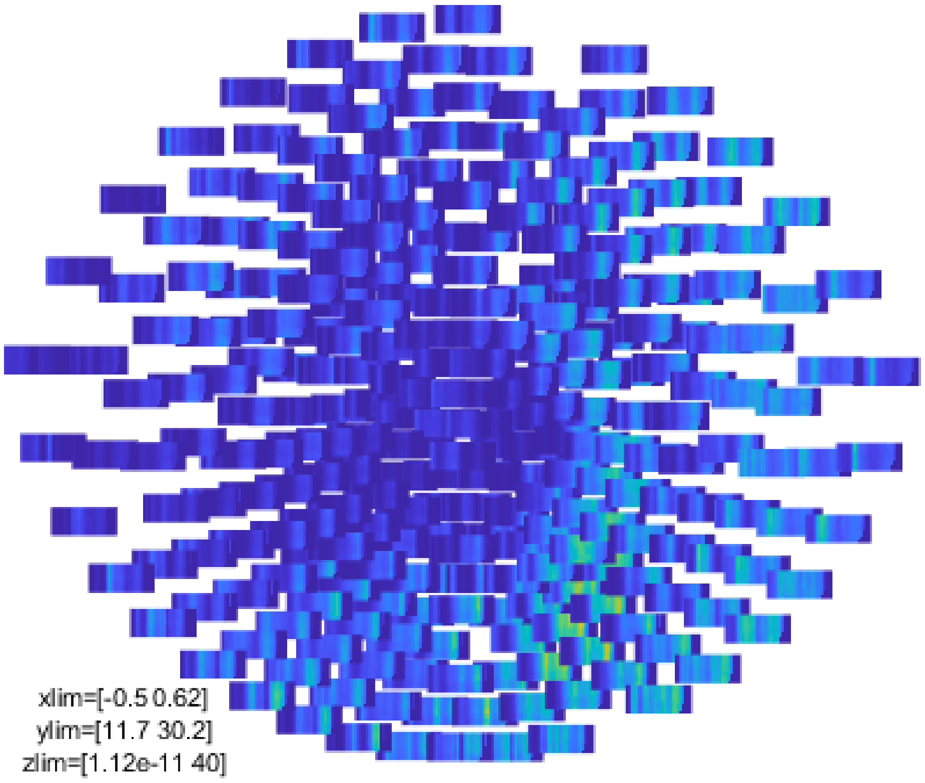

Figure S5. Time-frequency plots for individual MEG sensors for younger adults and trials with no perceived illusion. The x-axis denotes time from −500 ms to 620 ms, the y-axis denotes frequency, limited to the β-band (11.7 Hz to 30.2 Hz).

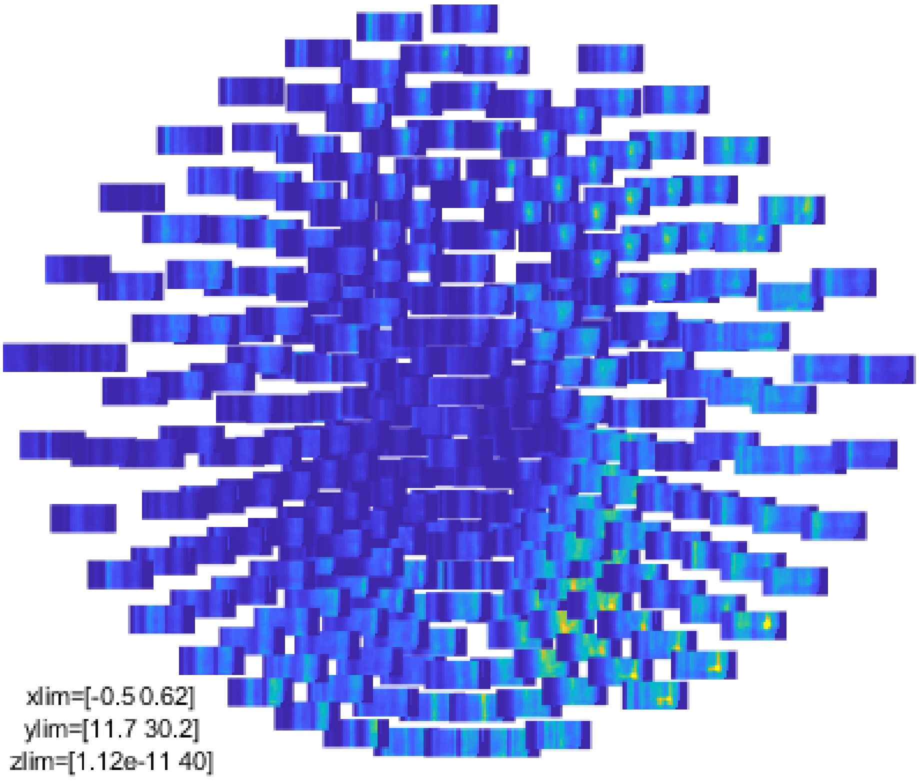

Figure S6. Time-frequency plots for individual MEG sensors for older adults and trials with perceived illusion. The x-axis denotes time from −500 ms to 620 ms, the y-axis denotes frequency, limited to the β-band (11.7 Hz to 30.2 Hz).

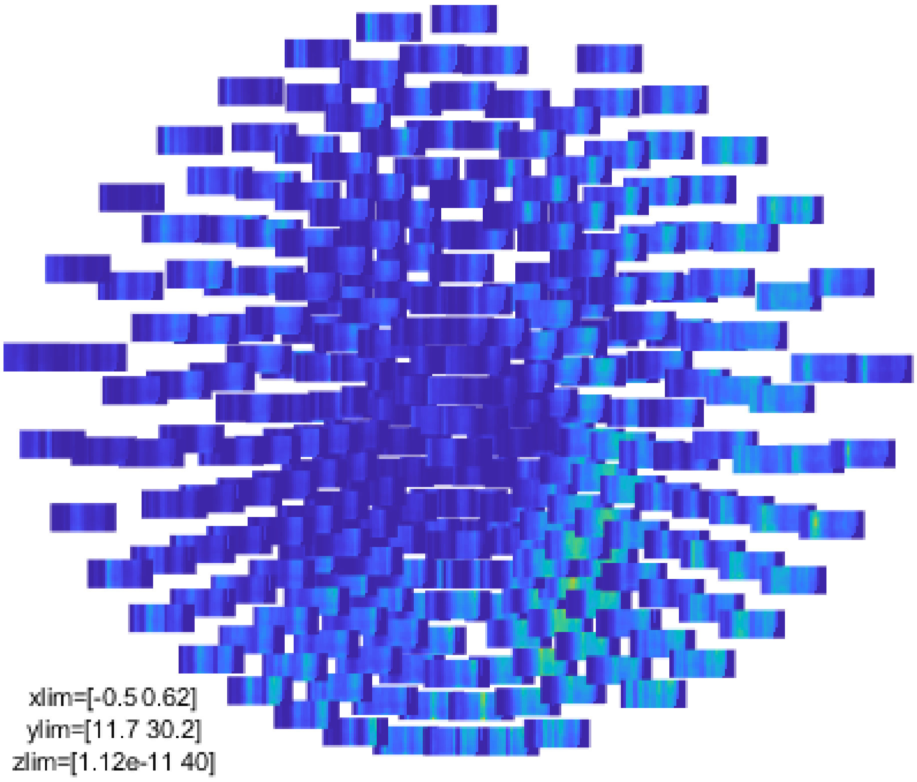

Figure S7. Time-frequency plots for individual MEG sensors for older adults and trials with no perceived illusion. The x-axis denotes time from −500 ms to 620 ms, the y-axis denotes frequency, limited to the β-band (11.7 Hz to 30.2 Hz).

Model 1 (All links)

Source

Target		MTG	MFG	Fusiform		BA22		BA18	
		Right	Right	Left	Right	Left	Right	Left	Right
MTG	Right				X		X		X
MFG	Right			X	X				
Fusiform	Left		X		X	X	X	X	X
	Right			X		X			X
BA22	Left			X	X		X	X	
	Right	X				X		X	
BA18	Left				X				X
	Right						X	X	

Model 2

Source

Target		MTG	MFG	Fusiform		BA22		BA18	
		Right	Right	Left	Right	Left	Right	Left	Right
MTG	Right				X		X		X
MFG	Right			X	O				
Fusiform	Left		X		X	X	X	X	X
	Right			X		X			X
BA22	Left			X	X		X	X	
	Right	X				X		X	
BA18	Left				X				X
	Right						X	X	

Model 3

Source

Target		MTG	MFG	Fusiform		BA22		BA18	
		Right	Right	Left	Right	Left	Right	Left	Right
MTG	Right				X		O		X
MFG	Right			X	X				
Fusiform	Left		X		X	X	X	X	X
	Right			X		X			X
BA22	Left			X	X		X	X	
	Right	X				X		X	
BA18	Left				X				X
	Right						X	X	

Model 4

Source

Target		MTG	MFG	Fusiform		BA22		BA18	
		Right	Right	Left	Right	Left	Right	Left	Right
MTG	Right				O		X		X
MFG	Right			X	X				
Fusiform	Left		X		X	X	X	X	X
	Right			X		X			X
BA22	Left			X	X		X	X	
	Right	X				X		X	
BA18	Left				X				X
	Right						X	X	

Model 5

Source

Target		MTG	MFG	Fusiform		BA22		BA18	
		Right	Right	Left	Right	Left	Right	Left	Right
MTG	Right				X		X		O
MFG	Right			X	X				
Fusiform	Left		X		X	X	X	X	X
	Right			X		X			X
BA22	Left			X	X		X	X	
	Right	X				X		X	
BA18	Left				X				X
	Right						X	X	

Model 6

Source

Target		MTG	MFG	Fusiform		BA22		BA18	
		Right	Right	Left	Right	Left	Right	Left	Right
MTG	Right				X		X		X
MFG	Right			X	X				
Fusiform	Left		X		X	X	X	X	X
	Right			X		X			O
BA22	Left			X	X		X	X	
	Right	X				X		X	
BA18	Left				X				X
	Right						X	X	

Model 7

Source

Target		MTG	MFG	Fusiform		BA22		BA18	
		Right	Right	Left	Right	Left	Right	Left	Right
MTG	Right				X		X		X
MFG	Right			X	X				
Fusiform	Left		X		X	X	X	X	X
	Right			X		X			X
BA22	Left			X	X		X	X	
	Right	X				X		X	
BA18	Left				X				X
	Right						O	X	

Model 8

Source

Target		MTG	MFG	Fusiform		BA22		BA18	
		Right	Right	Left	Right	Left	Right	Left	Right
MTG	Right				X		X		X
MFG	Right			X	X				
Fusiform	Left		X		X	X	X	X	X
	Right			X		X			X
BA22	Left			X	X		X	X	
	Right	X				X		X	
BA18	Left				O				X
	Right						X	X	

Model 9

Source

Target		MTG	MFG	Fusiform		BA22		BA18	
		Right	Right	Left	Right	Left	Right	Left	Right
MTG	Right				X		X		X
MFG	Right			X	X				
Fusiform	Left		X		X	X	X	X	O
	Right			X		X			X
BA22	Left			X	X		X	X	
	Right	X				X		X	
BA18	Left				X				X
	Right						X	X	

Model 10

Source

Target		MTG	MFG	Fusiform		BA22		BA18	
		Right	Right	Left	Right	Left	Right	Left	Right
MTG	Right				X		X		X
MFG	Right			X	X				
Fusiform	Left		X		O	X	X	X	X
	Right			X		X			X
BA22	Left			X	X		X	X	
	Right	X				X		X	
BA18	Left				X				X
	Right						X	X	

Model 11

Source

Target		MTG	MFG	Fusiform		BA22		BA18	
		Right	Right	Left	Right	Left	Right	Left	Right
MTG	Right				X		X		X
MFG	Right			X	X				
Fusiform	Left		X		X	X	X	X	X
	Right			X		X			X
BA22	Left			X	X		O	X	
	Right	X				X		X	
BA18	Left				X				X
	Right						X	X	

Model 12

Source

Target		MTG	MFG	Fusiform		BA22		BA18	
		Right	Right	Left	Right	Left	Right	Left	Right
MTG	Right				X		X		X
MFG	Right			X	X				
Fusiform	Left		X		X	X	O	X	X
	Right			X		X			X
BA22	Left			X	X		X	X	
	Right	X				X		X	
BA18	Left				X				X
	Right						X	X	

Model 13

Source

Target		MTG	MFG	Fusiform		BA22		BA18	
		Right	Right	Left	Right	Left	Right	Left	Right
MTG	Right				X		X		X
MFG	Right			X	X				
Fusiform	Left		X		X	X	X	X	X
	Right			X		O			X
BA22	Left			X	X		X	X	
	Right	X				X		X	
BA18	Left				X		X		X
	Right						X	X	

Model 14

Source

Target		MTG	MFG	Fusiform		BA22		BA18	
		Right	Right	Left	Right	Left	Right	Left	Right
MTG	Right				X		X		X
MFG	Right			X	X				
Fusiform	Left		X		X	X	X	X	X
	Right			X		X			X
BA22	Left			X	X		X	O	
	Right	X				X		X	
BA18	Left				X				X
	Right						X	X	

Model 15

Source

Target		MTG	MFG	Fusiform		BA22		BA18	
		Right	Right	Left	Right	Left	Right	Left	Right
MTG	Right				X		X		X
MFG	Right			O	X				
Fusiform	Left		X		X	X	X	X	X
	Right			X		X			X
BA22	Left			X	X		X	X	
	Right	X				X		X	
BA18	Left				X				X
	Right						X	X	

Figure S8. Tables of the links (“X”) that were included in each model. Model 1 contains all the links in the combined (illusion and no illusion) transfer entropy model. Models 25 and 26 represent the illusion-only and no illusion-only models from the transfer entropy analyses. For models 2 to 24, one link was removed. For the sake of clarity, “O” represent the missing links in each model. The winning model was model 5.

Model 16

Source

Target		MTG	MFG	Fusiform		BA22		BA18	
		Right	Right	Left	Right	Left	Right	Left	Right
MTG	Right				X		X		X
MFG	Right			X	X				
Fusiform	Left		O		X	X	X	X	X
	Right			X		X			X
BA22	Left			X	X		X	X	
	Right	X				X		X	
BA18	Left				X				X
	Right						X	X	

Model 17

Source

Target		MTG	MFG	Fusiform		BA22		BA18	
		Right	Right	Left	Right	Left	Right	Left	Right
MTG	Right				X		X		X
MFG	Right			X	X				
Fusiform	Left		X		X	X	X	X	X
	Right			X		X			X
BA22	Left			X	X		X	X	
	Right	O				X		X	
BA18	Left				X				X
	Right						X	X	

Model 18

Source

Target		MTG	MFG	Fusiform		BA22		BA18	
		Right	Right	Left	Right	Left	Right	Left	Right
MTG	Right				X		X		X
MFG	Right			X	X				
Fusiform	Left		X		X	X	X	O	X
	Right			X		X			X
BA22	Left			X	X		X	X	
	Right	X				X		X	
BA18	Left				X				X
	Right						X	X	

Model 19

Source

Target		MTG	MFG	Fusiform		BA22		BA18	
		Right	Right	Left	Right	Left	Right	Left	Right
MTG	Right				X		X		X
MFG	Right			X	X				
Fusiform	Left		X		X	O	X	X	X
	Right			X		X			X
BA22	Left			X	X		X	X	
	Right	X				X		X	
BA18	Left				X				X
	Right						X	X	

Model 20

Source

Target		MTG	MFG	Fusiform		BA22		BA18	
		Right	Right	Left	Right	Left	Right	Left	Right
MTG	Right				X		X		X
MFG	Right			X	X				
Fusiform	Left		X		X	X	X	X	X
	Right			X		X			X
BA22	Left			O	X		X	X	
	Right	X				X		X	
BA18	Left				X				X
	Right						X	X	

Model 21

Source

Target		MTG	MFG	Fusiform		BA22		BA18	
		Right	Right	Left	Right	Left	Right	Left	Right
MTG	Right				X		X		X
MFG	Right			X	X				
Fusiform	Left		X		X	X	X	X	X
	Right			O		X			X
BA22	Left			X	X		X	X	
	Right	X				X		X	
BA18	Left				X				X
	Right						X	X	

Model 22

Source

Target		MTG	MFG	Fusiform		BA22		BA18	
		Right	Right	Left	Right	Left	Right	Left	Right
MTG	Right				X		X		X
MFG	Right			X	X				
Fusiform	Left		X		X	X	X	X	X
	Right			X		X			X
BA22	Left			X	O		X	X	
	Right	X				X		X	
BA18	Left				X				X
	Right						X	X	

Model 23

Source

Target		MTG	MFG	Fusiform		BA22		BA18	
		Right	Right	Left	Right	Left	Right	Left	Right
MTG	Right				X		X		X
MFG	Right			X	X				
Fusiform	Left		X		X	X	X	X	X
	Right			X		X			X
BA22	Left			X	X		X	X	
	Right	X				X		O	
BA18	Left				X				X
	Right						X	X	

Model 24

Source

Target		MTG	MFG	Fusiform		BA22		BA18	
		Right	Right	Left	Right	Left	Right	Left	Right
MTG	Right				X		X		X
MFG	Right			X	X				
Fusiform	Left		X		X	X	X	X	X
	Right			X		X			X
BA22	Left			X	X		X	X	
	Right	X				X		X	
BA18	Left				X				O
	Right						X	X	

Model 25 (illusion-only)

Source

Target		MTG	MFG	Fusiform		BA22		BA18	
		Right	Right	Left	Right	Left	Right	Left	Right
MTG	Right				X		X		0
MFG	Right			X	X				
Fusiform	Left		X		0	0	0	0	X
	Right			0		0			X
BA22	Left			0	0		X	0	
	Right	O				X		X	
BA18	Left				X				X
	Right						0	X	

Model 26 (No illusion-only)

Source

Target		MTG	MFG	Fusiform		BA22		BA18	
		Right	Right	Left	Right	Left	Right	Left	Right
MTG	Right				0		0		X
MFG	Right			0	0				
Fusiform	Left		0		X	0	X	0	0
	Right			0		X			0
BA22	Left			0			X	X	
	Right	0				X		0	
BA18	Left				0				X
	Right						X	X	

Figure S9. Tables of the links included in each model (continued).

Model 1

Target		MTG Right	MFG Right	Fusiform Left	Fusiform Right	BA22 Left	BA22 Right	BA18 Left	BA18 Right
MTG	Right				X		X		X
MFG	Right			X	X				
Fusiform	Left		X		X	O	X	X	X
Fusiform	Right			X		X			X
BA22	Left			O	O		X	X	
BA22	Right	X				X		X	
BA18	Left				X				X
BA18	Right						X	X	

Model 2

Target		MTG Right	MFG Right	Fusiform Left	Fusiform Right	BA22 Left	BA22 Right	BA18 Left	BA18 Right
MTG	Right				X		X		X
MFG	Right			X	X				
Fusiform	Left		X		X	O	X	X	X
Fusiform	Right			O		X			X
BA22	Left			O	X		X	X	
BA22	Right	X				X		X	
BA18	Left				X				X
BA18	Right						X	X	

Model 3

Target		MTG Right	MFG Right	Fusiform Left	Fusiform Right	BA22 Left	BA22 Right	BA18 Left	BA18 Right
MTG	Right				X		X		X
MFG	Right			X	X				
Fusiform	Left		X		X	O	X	X	X
Fusiform	Right			O		X			X
BA22	Left			O	O		X	X	
BA22	Right	X				X		X	
BA18	Left				X				X
BA18	Right						X	X	

Model 4

Target		MTG Right	MFG Right	Fusiform Left	Fusiform Right	BA22 Left	BA22 Right	BA18 Left	BA18 Right
MTG	Right				X		X		X
MFG	Right			X	X				
Fusiform	Left		X		X	X	X	O	X
Fusiform	Right			O		X			X
BA22	Left			X	O		X	X	
BA22	Right	X				X		X	
BA18	Left				X				X
BA18	Right						X	X	

Model 5

Target		MTG Right	MFG Right	Fusiform Left	Fusiform Right	BA22 Left	BA22 Right	BA18 Left	BA18 Right
MTG	Right				X		X		X
MFG	Right			X	X				
Fusiform	Left		X		X	X	X	O	X
Fusiform	Right			O		X			X
BA22	Left			O	O		X	X	
BA22	Right	X				X		X	
BA18	Left				X				X
BA18	Right						X	X	

Model 6

Target		MTG Right	MFG Right	Fusiform Left	Fusiform Right	BA22 Left	BA22 Right	BA18 Left	BA18 Right
MTG	Right				X		X		X
MFG	Right			X	X				
Fusiform	Left		X		X	O	X	O	X
Fusiform	Right			X		X			X
BA22	Left			X	O		X	X	
BA22	Right	X				X		X	
BA18	Left				X				X
BA18	Right						X	X	

Model 7

Target		MTG Right	MFG Right	Fusiform Left	Fusiform Right	BA22 Left	BA22 Right	BA18 Left	BA18 Right
MTG	Right				X		X		X
MFG	Right			X	X				
Fusiform	Left		X		X	O	X	O	X
Fusiform	Right			O		X			X
BA22	Left			X	O		X	X	
BA22	Right	X				X		X	
BA18	Left				X				X
BA18	Right						X	X	

Model 8

Target		MTG Right	MFG Right	Fusiform Left	Fusiform Right	BA22 Left	BA22 Right	BA18 Left	BA18 Right
MTG	Right				X		X		X
MFG	Right			X	X				
Fusiform	Left		X		X	O	X	O	X
Fusiform	Right			X		X			X
BA22	Left			O	O		X	X	
BA22	Right	X				X		X	
BA18	Left				X				X
BA18	Right						X	X	

Model 9

Target		MTG Right	MFG Right	Fusiform Left	Fusiform Right	BA22 Left	BA22 Right	BA18 Left	BA18 Right
MTG	Right				X		X		X
MFG	Right			X	X				
Fusiform	Left		X		X	O	X	O	X
Fusiform	Right			O		X			X
BA22	Left			O	X		X	X	
BA22	Right	X				X		X	
BA18	Left				X				X
BA18	Right						X	X	

Model 10

Target		MTG Right	MFG Right	Fusiform Left	Fusiform Right	BA22 Left	BA22 Right	BA18 Left	BA18 Right
MTG	Right				X		X		X
MFG	Right			X	X				
Fusiform	Left		X		X	O	X	O	X
Fusiform	Right			O		X			X
BA22	Left			O	O		X	X	
BA22	Right	X				X		X	
BA18	Left				X				X
BA18	Right						X	X	

Model 11

Target		MTG Right	MFG Right	Fusiform Left	Fusiform Right	BA22 Left	BA22 Right	BA18 Left	BA18 Right
MTG	Right				X		X		X
MFG	Right			X	X				
Fusiform	Left		X		X	O	X	X	X
Fusiform	Right			X		X			X
BA22	Left			O	O		X	X	
BA22	Right	O				X		X	
BA18	Left				X				X
BA18	Right						X	X	

Model 12

Target		MTG Right	MFG Right	Fusiform Left	Fusiform Right	BA22 Left	BA22 Right	BA18 Left	BA18 Right
MTG	Right				X		X		X
MFG	Right			X	X				
Fusiform	Left		X		X	O	X	X	X
Fusiform	Right			O		X			X
BA22	Left			O	X		X	X	
BA22	Right	O				X		X	
BA18	Left				X				X
BA18	Right						X	X	

Model 13

Target		MTG Right	MFG Right	Fusiform Left	Fusiform Right	BA22 Left	BA22 Right	BA18 Left	BA18 Right
MTG	Right				X		X		X
MFG	Right			X	X				
Fusiform	Left		X		X	O	X	X	X
Fusiform	Right			O		X			X
BA22	Left			O	O		X	X	
BA22	Right	O				X		X	
BA18	Left				X				X
BA18	Right						X	X	

Model 14

Target		MTG Right	MFG Right	Fusiform Left	Fusiform Right	BA22 Left	BA22 Right	BA18 Left	BA18 Right
MTG	Right				X		X		X
MFG	Right			X	X				
Fusiform	Left		X		X	X	X	O	X
Fusiform	Right			O		X			X
BA22	Left			X	O		X	X	
BA22	Right	O				X		X	
BA18	Left				X				X
BA18	Right						X	X	

Model 15

Target		MTG Right	MFG Right	Fusiform Left	Fusiform Right	BA22 Left	BA22 Right	BA18 Left	BA18 Right
MTG	Right				X		X		X
MFG	Right			X	X				
Fusiform	Left		X		X	X	X	O	X
Fusiform	Right			O		X			X
BA22	Left			O	O		X	X	
BA22	Right	O				X		X	
BA18	Left				X				X
BA18	Right						X	X	

Model 16

Target		MTG Right	MFG Right	Fusiform Left	Fusiform Right	BA22 Left	BA22 Right	BA18 Left	BA18 Right
MTG	Right				X		X		X
MFG	Right			X	X				
Fusiform	Left		X		X	O	X	O	X
Fusiform	Right			X		X			X
BA22	Left			X	O		X	X	
BA22	Right	O				X		X	
BA18	Left				X				X
BA18	Right						X	X	

Model 17

Target		MTG Right	MFG Right	Fusiform Left	Fusiform Right	BA22 Left	BA22 Right	BA18 Left	BA18 Right
MTG	Right				X		X		X
MFG	Right			X	X				
Fusiform	Left		X		X	O	X	O	X
Fusiform	Right			O		X			X
BA22	Left			X	O		X	X	
BA22	Right	O				X		X	
BA18	Left				X				X
BA18	Right						X	X	

Model 18

Target		MTG Right	MFG Right	Fusiform Left	Fusiform Right	BA22 Left	BA22 Right	BA18 Left	BA18 Right
MTG	Right				X		X		X
MFG	Right			X	X				
Fusiform	Left		X		X	O	X	O	X
Fusiform	Right			X		X			X
BA22	Left			O	O		X	X	
BA22	Right	O				X		X	
BA18	Left				X				X
BA18	Right						X	X	

Figure S10. A separate set of DCM models were created to remove cascades and common drive artifacts. “X”’s represent the links in each model; “O”’s represent the missing links in each model. Models 11 to 20 are replications of models 1 to 10, respectively; with the exception that with each model, the link between the right middle temporal gyrus and right BA22 was also removed.

Model 19

Target		Source: MTG	MFG	Fusiform		BA22		BA18	
		Right	Right	Left	Right	Left	Right	Left	Right
MTG	Right				X		X		X
MFG	Right			X	X				
Fusiform	Left		X		X	O	X	O	X
	Right			O		X			X
BA22	Left			O	X		X	X	
	Right	O				X		X	
BA18	Left				X				X
	Right						X	X	

Model 20

Target		Source: MTG	MFG	Fusiform		BA22		BA18	
		Right	Right	Left	Right	Left	Right	Left	Right
MTG	Right				X		X		X
MFG	Right			X	X				
Fusiform	Left		X		X	O	X	O	X
	Right			O		X			X
BA22	Left			O	O		X	X	
	Right	O				X		X	
BA18	Left				X				X
	Right						X	X	

Figure S11. Tables of the links included in each model (continued).
